# 3D Printing of pH Indicator Auxetic Hydrogel Skin Wound Dressing

**DOI:** 10.3390/molecules28031339

**Published:** 2023-01-31

**Authors:** Filmon Tsegay, Muhammed Hisham, Mohamed Elsherif, Andreas Schiffer, Haider Butt

**Affiliations:** Department of Mechanical Engineering, Khalifa University, Abu Dhabi 127788, United Arab Emirates

**Keywords:** hydrogel wound dressings, PAA adhesion, pH sensor, 3D printing, auxetic structures

## Abstract

The benefits of enclosing pH sensors into wound dressings include treatment monitoring of wounded skin and early detection of developing chronic conditions, especially for diabetic patients. A 3D printed re-entrant auxetic hydrogel wound dressing, doped with pH indicator phenol red dye, was developed and characterized. The re-entrant auxetic design allows wound dressing adhesion to complex body parts, such as joints on arms and legs. Tensile tests revealed a yield strength of 140 kPa and Young’s modulus of 78 MPa. In addition, the 3D-printed hydrogel has a swelling capacity of up to 14%, limited weight loss to 3% in six days, and porosity of near 1.2%. A reasonable pH response resembling human skin pH (4–10) was obtained and characterized. The integration of color-changing pH indicators allows patients to monitor the wound’s healing process using a smartphone. In addition to the above, the mechanical properties and their dependence on post-processing were studied. The results show that the resin composition and the use of post-treatments significantly affect the quality and durability of the wound dressings. Finally, a poly (acrylic acid) (PAA) and water-based adhesive was developed and used to demonstrate the performance of the auxetic wound dressing when attached to moving body joints.

## 1. Introduction

Skin is the largest organ in the human body, and one of its functions is to defend the body against external, chemical, and biological pathogens [1]. Additionally, it prevents water loss from the body (dehydration) and regulates temperature [1]. The skin pH is acidic in the range of 4–6 under normal conditions, and when the skin is under chronic wounds, the pH was found to lie in the range of 7.5–8.9 [2]. Healthy skin has a weakly acidic pH, which assists in combating harmful microbes and damaging free radicals that can accelerate the aging process. However, human skin increases its pH if exposed to harsh environments (e.g., air pollution and humidity levels), antibacterial soaps and gels, sweat, longtime exposure to the sun, and injuries [1]. The main reason for changing the skin pH from the acidic to alkaline level is the release of exudate from the peripheral tissues to the wound site. If the skin is too alkaline, the skin will look flaky and red, and if it is too acidic, it increases the chance of inflammatory skin conditions such as eczema and acne [3]. Therefore, monitoring the skin pH is crucial for early detection of acidic behavior and the prevention of inflammatory wound conditions.

Hydrogels are classified as smart materials that can respond to physical stimuli, such as temperature, electric and magnetic fields, light intensity, and pressure [1]. Additionally they can be functionalized to respond to chemical stimuli, such as pH, ions, and specific chemical compositions [1,4]. Their response to pH sensing can be either by color or volumetric changes. The fascinating feature of hydrogels is their ability to return to their original volume once the pH triggers are removed [4]. Transducers are typically used to monitor the volumetric response of pH-responsive hydrogels [1]. The transducers track the changes based on two principles: (i) mechanical work induced by the change of the volume that can be tracked using a micro-cantilever bending plate transducer, and (ii) observation of changes in properties, such as optical color, conductance, and oscillation mechanisms [1]. Immersing the hydrogel in different pH solutions leads to a change in the quantity of dissociated carboxylic ions that alter the volume and refractive index of the hydrogel [5]. Generally, upon swelling of the hydrogels, the refractive index undergoes a negative shift, as pure water usually possesses a lower refractive index than the hydrogel in its unhydrated condition [6]. Tomar et al. [7] analyzed the swelling of pH-responsive particles in response to several pH ranges, and observed that at pH 7.4, water uptake occurred.

However, when hydrogels are 3D printed via stereolithography-based techniques, such as digital light processing (DLP), they present a less pronounced volumetric response towards pH changes. The main reason could be that the photopolymerization of the hydrogel hinders the extracellular matrix (ECM) from further enlargement. Several studies have reported the incorporation of pH sensors into hydrogel-based wound dressings following their fabrication via 3D printing. External pH biosensors (i.e., either biomarkers or electronics) integrated in hydrogel wound dressings should be similarly flexible and stretchable as the hydrogel matrix and body contours, while also being biocompatible and non-toxic [1]. It is also desirable that they respond to potential infections or hyper-inflammation in chronic wounds [8], and their degradation rate should be proportional to the degradation of the hydrogel matrix. The major challenges in integrating separate bio-sensors into hydrogel wound dressings include the complex design process involved, and the added costs of fabrication and integration. To overcome the limitations of external pH biosensors in wound dressing applications, some researchers have explored the possibility of using cost-effective and non-cytotoxic dyes. One of the most common dyes used in biomedical applications is phenol red. For example, Liu et al. [3] reported hydrogel wound bandages doped with phenol red dyes that can sense pH variations in the wound over a range of 5 to 9, which relates to common pH ranges of the skin. El-Nahhal et al. [9] reported a 3D printed wound dressing dipped inside phenol red solution to entrap the dye inside a hydrogel matrix. Phenol red was trapped into different silica hydrogels in the presence of ethanediyl-1, 2-bis (dimethyldodecylammonium bromide) (Gemini 12–2-12), alkyl hydroxyethyl dimethyl ammonium chloride (HY, R= 12–14), and sodium dodecyl sulfate (SDS) surfactants [9]. The colorimetric appearance of the hydrogel changed with pH, and the absorption peak positions were correlated to the pH ranges. However, these studies [2,10,11,12,13,14] did not disclose the drainage capability of the dyes from the hydrogel matrix. This is an important aspect because the entrapment only occurred on the surface of the wound dressing, which causes dye leakage when they come in contact with excess exudates. The main reason for the drainage could be that less breakdown in organic solutions results in more subordinate diffusion and non-bond surface entrapment of the dyes. As a result, the solution dyes are impeded from diffusing inside the ECM of the hydrogel.

Another important aspect in the development of hydrogel wound dressings is their adhesion capability. Several studies have shown that the integration of poly (acrylic acid) (PAA) in hydrogels can provide an effective adhesion mechanism. For example, F. Tsegay et al. [10] developed a smart auxetic hydrogel wound dressings using monomers of 2-Hydroxyethyl methacrylate (HEMA) as hydrogel, and photoinitiator of diphenyl-(2,4,6-trimethyl benzoyl)phosphine oxide (TPO) and poly(ethylene glycol) dimethacrylate (PEGDA) as crosslinker. The result has shown a significant tensile strength and high absorption rate of fluids of up to 30%. The main reason behind the result could be the single crosslinking nature of the HEMA-based hydrogel. Thomas et al. [11] developed a 3D printed self-adhesive polyethylene glycol dimethacrylate C_10_H_14_O_4_ and poly(acrylic acid) (PEGDA-PAA) hydrogel as a modular component for soft actuators. They snapped the printed hydrogel and reattached it to the body’s original form to test the PAA’s adhesion capability. Zhao et al. [12] additively manufactured a stretchable and highly adhesive Ti_3_C_2_Tx-PAA hydrogel using ink writing 3D printing. They demonstrated the adhesion capability on various materials, such as glass, metal, PTFE, and pigskin, with different concentrations of Ti_3_C_2_Tx and PAA, and it was found that increasing the concentration of PAA enhanced the adhesion strength significantly. Meinderink et al. [13] modified the surface morphology of ZnO nanostructure films and dipped them in dilute and an aqueous solution of PAA. In addition, Zhang et al. [14] examined the adhesion capability of PAA/NIPAAM-co-DMA hydrogels over a temperature range of 15–35 °C, reporting a shear strength of 550 kPa, 400 kPa, and 650 kPa at 15 °C, 25 °C, and 35 °C, respectively. In addition, Yang et al. [15] developed a 1D polymer of BCD/PDA/PAM hydrogels adhered to some of the body’s moving joints. They include brushes that have rigid bacterial cellulose (BC) backbones to enhance the mechanical properties of the hydrogel (tensile strength 21–51 kPa).

Here, we Introduce an alcohol-based mixing method for homogeneous integration of phenol red into the hydrogel matrix, since phenol red dyes dissolve more efficiently in ethanol than in water [16]. Specifically, a HEMA/PEGDA/TPO/AA-PR hydrogel resin was prepared in this study. Following 3D printing of the prepared resins via DLP, the physical properties of the 3D printed hydrogels were characterized, including optical pH color-changing behavior of phenol red in the pH range of 4 to 10. In addition, the shrinkage behavior, surface roughness, swelling capacity, weight loss, and porosity of the HEMA/PEGDA/TPO/AA-PR hydrogel were also characterized. Then, a re-entrant auxetic hydrogel-based wound dressing was designed and 3D printed via DLP. The emergence of additive manufacturing (AM) in biomedical applications significantly reduced the manufacturing time and enhanced the quality and functionality of the fabricated parts [17,18,19,20]. Re-entrant auxetic structures are metamaterials with tunable weight-specific strength and stiffness, and their design was chosen based on the comparative study by Andres et al. [17]. Moreover, re-entrant structures possess a negative Poisson’s ratio (i.e., auxetic behavior), which facilitates their attachment to body parts with complex curvatures, such as the knee or elbow. Finally, a PAA and water-based adhesive is developed to demonstrate the performance of the auxetic wound dressing when attached to moving body joints.

## 2. Results and Discussion

### 2.1. Optical Characterization of the 3D Printed HEMA/PEGDA/TPO/AA Hydrogels

**Figure 1:**
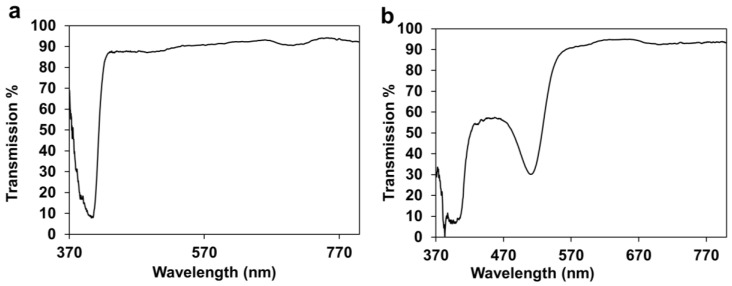
(**a**). Optical transmission of 3D printed *HEMA/PEGDA/TPO/AA*. (**b**) Optical transmission of 3D printed *HEMA/PEGDA/TPO/AA-PR*.

One of the benefits of HEMA hydrogel based wound dressings is their optical transparency, which makes it possible to monitor the healing process of the wound without removing the bandage from the skin [1]. The 3D printed HEMA/PEGDA/TPO/AA-PR wound dressings possessed reasonable transparency with a dip in the transmission observed around 510 nm (Figure 1a,b). The transmission of 3D printed hydrogel bandages with phenol red showed high transparency above the wavelength of 510 nm; however, due to the presence of the photo-initiator, its transmission was reduced in the UV region below 400 nm (Figure 1b). The results presented in Figure 1a,b demonstrate that the healing process of the wound can be monitored through visual inspection. The transparency of hydrogel has several advantages in wound healing, such as reducing inflammation and scarring of tissues, as compared to sutures [9]. In addition, E. Pawlowska et al. [21] studied the cytotoxicity and genotoxicity of 2-hydroxyethyl methacrylate (HEMA) in human peripheral blood lymphocytes and A549 lung-tumor cells. The result has shown that the HEMA at concentrations up to 10 mM neither affected the viability of the cells nor interacted with isolated plasmid DNA during a 1h exposure.

### 2.2. Shrinkage Behavior

Shrinkage behavior showed that the width, length, and thickness changed by 2.4%, 2.6%, and 1.23% respectively. The results were measured pre and post printing process. The main reason behind the expanded size could be the replacement of long-distance connections via the Van der Waals force by strong and short covalent bonds between the carbon atoms of different monomer units.

### 2.3. Fourier Transform Infrared Spectroscopy (FTIR) Analysis

**Figure 2:**
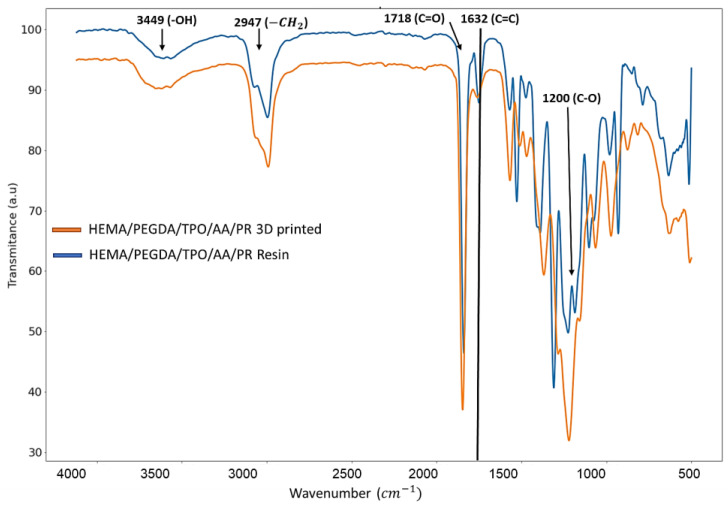
*FTIR charctaerzation of HEMA/PEGDA/TPO/AA/PR resin and 3D printed sample*.

In our study, FTIR was used to examine the chemical composion of the resin before and after the printing process. In experimental results, in the HEMA/PEGDA/TPO/AA/phenol red spectrum (Figure 2), there was an –OH bond at 3449 cm^−^^1^, a CH_2_ at 2947 cm^−^^1^, a C = O at 1718 cm^−^^1^, and a C = C at 1632 cm^−^^1^. In the 3D printing of the HEMA/PEGDA/TPO/AA/phenol red hydrogel spectrum, after the UV light crosslinking reaction, there was an –OH bond at 3418 cm^−^^1^, and the C = C bond at 1619 cm^−^^1^ had disappeared. The above result have been verified using the Yi-Huang Hsueh et al. [22] FTIR results that analyzed the HEMA-based hydrogel’s chemical composition and bonds of the monomers. In their findings of the HEMA spectrum, there was an –OH bond at 3500 cm^−^^1^, a CH_2_ at 2940 cm^−^^1^, a C = O at 1730 cm^−^^1^, and a C = C at 1632 cm^−^^1^. In addition, in the PEGDA spectrum, there was an –OH at 3600 cm^−^^1^, a CH_2_ at 2860 cm^−^^1^, a C = O at 1730 cm^−^^1^, and a C = C at 1632 cm^−^^1^ [22]. In the crosslinked hydrogel film spectrum, after the UV light crosslinking reaction, there was an –OH at 3400 cm^−^^1^, and the C = C at 163 cm^−^^1^ from PEGDA had vanished. TPO has –CH3 at 2963 cm^−^^1^ and has shown a minimal effect [22]. This suggests that the HEMA-based hydrogel is successfully crosslinked. It also shows that the UV light has curing effect on the HEMA-based hydrogel bond during 3D printing. Comparing to the above-mentioned peer reviewed paper, the outcome indicates that there is no considerable bond or crosslinking between the phenol red and the HEMA/PEGDA/TPO/AA. The phenol red was entrapped inside the HEMA/PEGDA/TPO/AA matrix in both the pre and post of 3D printing.

### 2.4. pH Characterization of the 3D Printed HEMA/PEGDA/TPO/AA Hydrogels

**Figure 3:**
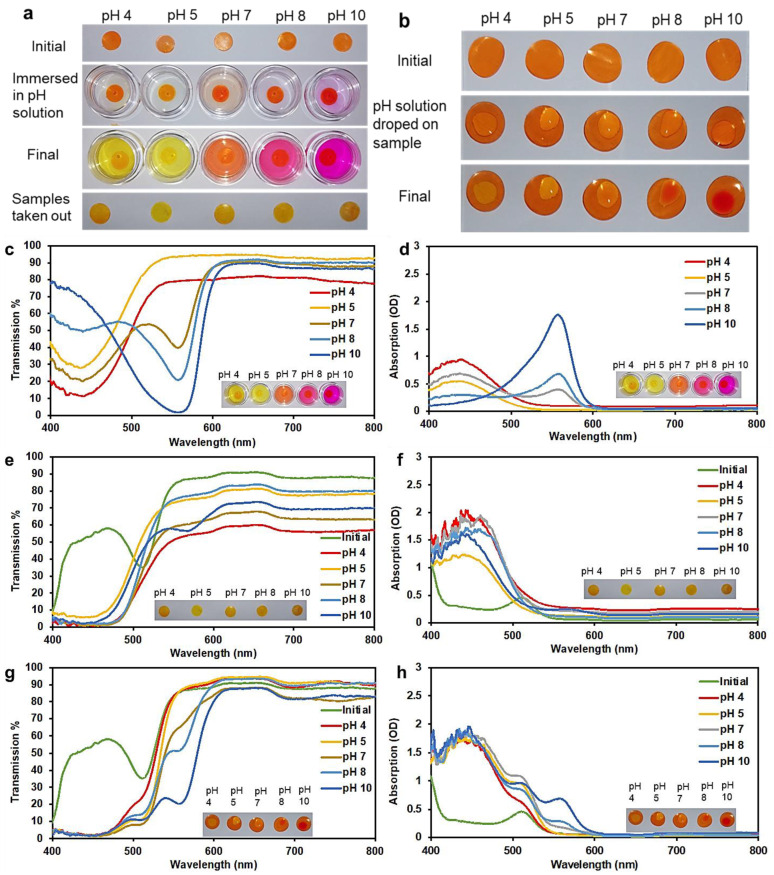
(**a**) The 3D printed hydrogels tested by immersion in buffer solutions of different pH. (**b**) The 3D printed hydrogels tested by dropping the pH solution onto the sample. (**c**) Optical transmission and (**d**) absorption from pH buffer solutions after hydrogel samples were immersed in them. (**e**) Optical transmission and (**f**) absorption from the hydrogel samples taken out after immersing in pH solutions. (**g**) Optical transmission and (**h**) absorption from hydrogel samples with the pH solution dropped on surface of dry 3D printed hydrogel.

When 3D printed hydrogels containing phenol red were immersed in pH solutions, the solutions changed color according to their pH (Figure 3a). The dye contained in the hydrogel slowly leaked into the pH solution and then changed its color (over a duration of 2–3 h). The hydrogel sample, on the other hand, retained almost the same color. When pH solutions were dropped onto hydrogel samples, these drops of liquid also slowly changed color (in 2–3 h) due to the phenol red that leaks into them from the sample (Figure 3b). In the liquid, from pH 4 to pH 5, the color turned more deeply yellow. At pH 7 the color became orange-red. At pH 8, the color became purple. At pH 10, the color turned deeply purple. Such changes in color were also clear in optical transmission and absorption spectra obtained using white light (Figure 3c–h). From pH 4 to 10, the absorption peak slowly changes from around 440 nm to 557 nm. From pH 4 to 5, the absorption at 440 nm decreased. Then at pH 7, a weak absorption peak appeared at 557 nm. From pH 8 to 10, the peak at 440 nm disappeared and the peak at 557 nm became more pronounced. In the initial dry state (Figure 3d), pH 8 has an absorption peak at 510 nm. In addition, in Figure 3d, at pH 8, a high saturation of the color was perceived as a result, and a sharp absorption peak was detected. In addition, a strong sharp absorption peak is noticed when a brilliant strong color is present. However, after immersion in liquid solutions, the latter peak disappeared and a peak appeared at 440 nm. This same change occurred for samples immersed in all pH solutions. The only exception was that at pH 10, an additional very minor absorption peak appeared at 557 nm. In Figure 3f, the positions of the peaks and dips were located at different wavelengths, in addition to the change in the width of both valleys and peaks. The peaks were centered on the wavelengths of 450, 455, 440, 460, and 455 nm, for pH concentrations of 4, 5, 7, 8, and 10 respectively, which confirms that the peak position red-shifts with high pH concentration. In addition, in Figure 3f these are distortions due to the spectrometer. The light source has a low intensity in the 400 to 500 range, which causes these artifacts in the spectra.

The almost identical trends at all pH levels are likely due to the effect of the pH of HEMA/PEGDA/TPO/AA. HEMA/PEGDA/TPO/AA itself has a *pKa* ≈ 5.2. In the dry state, the interaction between the dye and HEMA/PEGDA/TPO/AA is weak, so the dye shows its own original color. When the sample is immersed in a liquid, the sample absorbs some of this liquid and the dye interacts with HEMA/PEGDA/TPO/AA, showing the color that matches its pH (which is around 6). When the sample is placed in a pH solution, a small amount of pH solution is absorbed by the sample, but this absorbed liquid is a very small fraction compared to the amount of HEMA/PEGDA/TPO/AA. Effectively, the pH inside the sample is still close to the pH of HEMA/PEGDA/TPO/AA. A solution to overcome this issue would be to increase the swelling capacity of the hydrogel to a very high value, perhaps 100% or 200% [23]. Then it may be expected that the sample itself will show significant changes in color due to the higher fraction of pH solution absorbed by the sample. Since patients cannot use the spectrometer at home, they could detect the color range using a smartphone application [24,25,26].

### 2.5. Weight Loss (Degradation) of 3D Printed HEMA/PEGDA/TPO/AA Hydrogels

**Figure 4:**
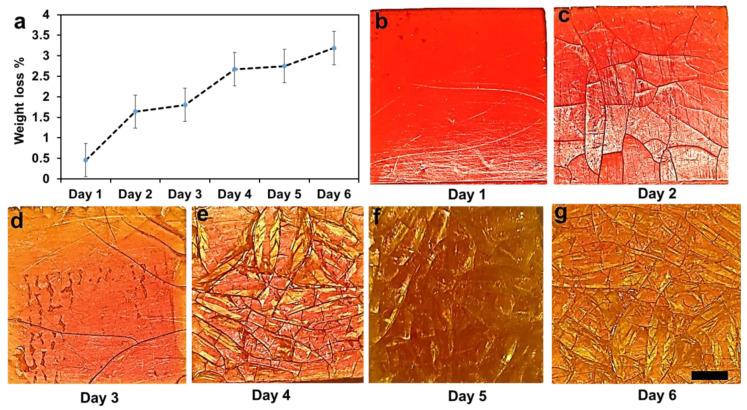
Weight loss of the composites up to six days. (**a**) Weight loss percentage over 6 days. (**b**–**g**) Degradation processes were carried out at room temperature (25 °C) in PBS. Scale bar 0.5 mm.

The biodegradation behavior of the 3D printed hydrogel was characterized by measuring its weight loss over six days (Figure 4). The results showed a steady weight loss over the test period, reaching around 3% at day 6 (Figure 4a). We also observed a significant change in the surface topography over time, as seen from Figure 4b–g. These degradation phenomena can be linked to various additives in the 3D printed hydrogel (particularly 10 wt.% of TPO and 45 wt.% of crosslinker), which contributed to a significant weight fraction of the 3D printed hydrogel. From the experiment, we observed the delamination of a surface layer, which eventually peeled off after 3 days (Figure 4d), hence changing the color of the surface. The degradation process then repeated itself until a new layer peeled off on day 5.

In hydrogel polymers, usually the lowest crosslink density can be degraded entirely under 24 h [27]; however, the highest crosslinked hydrogel can last longer due to increasing the molecular weight of the hydrogel. To validate our outcome, we compared it with an experiment conducted on degradable pHEMA hydrogels in enzymatic solutions by S. Atzet et al. [27]. The result shows that 30% mass loss in 16 weeks. Thus, they deduced that the degradable pHEMA developed in this study has considerable potential as a scaffold for tissue engineering in cardiac and other applications. In our *HEMA/PEGDA/TPO/AA hydrogel* study, the effect of photopolymerization on crosslinking is shown to be significant.

### 2.6. Swelling Behavior, Porosity and Surface Wettability of 3D Printed HEMA/PEGDA/TPO/AA Hydrogels

**Figure 5:**
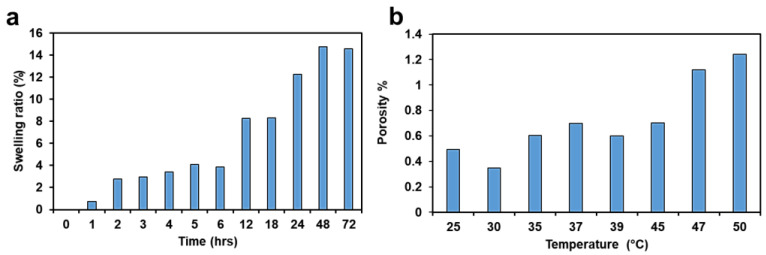
(**a**) Swelling ratio. (**b**) Porosity.

As shown in Figure 5a, the swelling ratio of the hydrogels increased with increasing soaking time, as expected. The swelling process briefly halted after 12 h, but then the swelling ratio further grew by 4% between 18 and 24 h. After 72 h, a final swelling ratio of ≈14% was reported. The results demonstrate that the 3D printed hydrogel wound dressing has an excellent capability to absorb a moderate wound exudate or can deliver fluids to any dry wound when needed [10]. The main reason behind the high absorption rate could be the nature of the hydrogel. Hydrogels are very flexible in a similar way to human tissues because of the considerable water content [28]. The 3D printed hydrogel still keeps the hydrophilicity of a hydrogel network that results from the hydrophilic functional groups distributed in the structure, such as hydroxyl, carboxylic, amine, sulphonyl hydroxide, and amide groups [1].

As shown in Figure 5b, the porosity of the hydrogel increased with an increase in temperature. The physical interpretation of this result is that the permeable fiber in the 3D printed hydrogel sample consists of three-dimensional layers of components bonded together. This creates a void between the fibers to hold fluid, making them porous [29]. The main reason behind the 25 °C could be the presence of unstable monomers. The freshly 3D printed sample has more void during taken off from the printing beds. However, as the sample is at ambient temperature then the printed layers come to stable condition, therefore some of the voids are filled. However, as the temperature increases, the porosity increases, since the filled voids are expanded. When the temperature increases from 30 °C to 50 °C, the fibers of the polymer network expand, and the sizes of the pores grow. These results suggest that 3D printed hydrogels can be used for temperature-controlled delivery of drugs in various biomedical applications [30]. The SD of the swelling ratio and the porosity are 3.53 and 0.28, respectively

Surface wettability quantifies the level of wetting when solid and liquid phases interact with each other, and is measured by contact angles (CA) [31]. A smaller contact angle (CA < 90°) infers a higher surface wettability, whereas a higher contact angle (CA > 90°) implies lower surface wettability [31]. Surface wettability correlates with surface roughness, as roughness creates additional surface area that can make adhesive contact when forming a bond with the liquid. The average CA was 81.75° for UV post-cured samples, with a SD of 2.13. The CA of *HEMA/PEGDA/TPO/AA* hydrogel is more hydrophilic when compared to the common PEGDMA and 70% of HEMA-based hydrogel that have the lowest contact angle of 98° [30].

### 2.7. Mechanical Property Analysis of 3D Printed HEMA/PEGDA/TPO/AA Hydrogels

#### 2.7.1. Effect of Resin Composition on the Mechanical Properties

**Figure 6:**
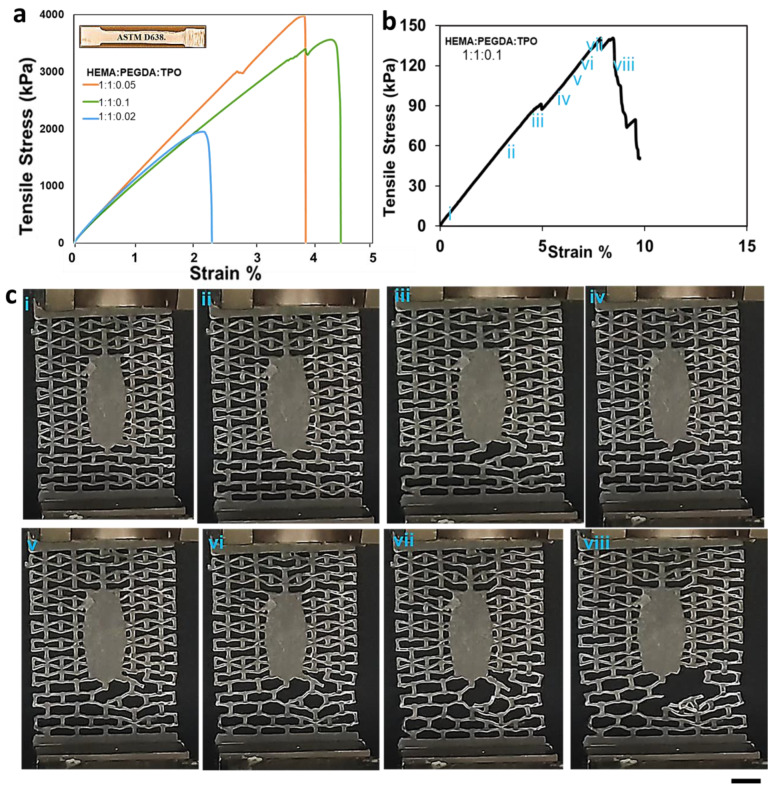
(**a**) Tensile stress–strain curves for 3D printed hydrogels with different HEMA: PEGDA: TPO concentrations. (**b**) The mechanical strength corresponding to the re-entrant unit cell fail representation using stress and strain diagram.(i-vii) the elastic deformation section (viii) failure (**c**) Failing stage of the re-entrant unit cells. (i-viii) The response of the unit cells to the applied load. Scale 0.5 cm.

The effect of the concentration of cross-linkers, monomers, and photoinitiators on the mechanical properties of hydrogels has been thoroughly discussed in the literature [32,33,34,35,36,37,38,39]. It has been established that a higher proportion of the cross-linker relative to the functional monomer can increase the strength and hardness of the polymer [40] while reducing its swelling ratio. In this study, we examined the effect of the HEMA: PEGDA: TPO ratio on the tensile response of the as-printed hydrogel. When the latter ratio was 0.5:1:0.1 or 1:0.5:0.1, the samples could not be removed from the print bed without fracturing them, as a result of a poor balance between the HEMA monomer and the PEGDA cross-linker. The main reason behind the ratios is a high cross-linking concentration increases the molecular weight and impedes the mobility of the monomers; as a result, glassy behavior has been shown during the 3D printing process. When the concentration decreases, it has the opposite effect, and fails to be 3D printed. Successful prints could only be obtained with identical concentrations of monomer and cross-linker, as seen from Table 1. In the latter case, the effect of increasing the concentration of the TPO photo-initiator from 0.02 to 0.05 wt.% is to increase both the tensile strength and failure strain of the hydrogel (Figure 6a). A higher amount of photo-initiator produces more radicals during photopolymerization and helps in reducing the number of unreacted monomers. However, as the TPO concentration was further increased to 0.1 wt.%, the tensile strength slightly decreased, but this came with the benefit of enhanced strain tolerance (i.e., higher failure strain). Hence, the composition HEMA: PEGDA: TPO = 1:1:0.1 was chosen for fabricating the auxetic wound dressing, since it achieved the best balance between strength and stretchability. For the study purpose, a triplicate study was performed, and the calculated SD is 1.86.

#### 2.7.2. Mechanical Response of the Auxetic Hydrogel Wound Dressing

Once the optimized resin composition (HEMA: PEGDA: TPO = 1:1:0.1) were established, an auxetic wound dressing with re-entrant topology was 3D printed and tested in uniaxial tension. Following 3D printing, the wound dressings were UV post-cured for 3 min. As shown in Figure 6c, the response of the wound dressing was linear elastic up to a strain of 5%. During this phase of the response, the deformation primarily occurred in the bottom portion of the patch outside the elliptic wound cover (Figure 6c). As the deformation increased, the localization of deformation in the bottom part became more pronounced, giving rise to localized failure of some struts near the elliptic wound cover at a strain of 5% and stress of 80 kPa. However, ultimate failure of the wound dressing occurred later, at a strain of 8%, reporting an ultimate strength of 140 kPa, which corresponds to a force of 3.7 N.

### 2.8. Adhesion Capability of 3D Printed HEMA/PEGDA/TPO/AA Hydrogels

**Figure 7:**
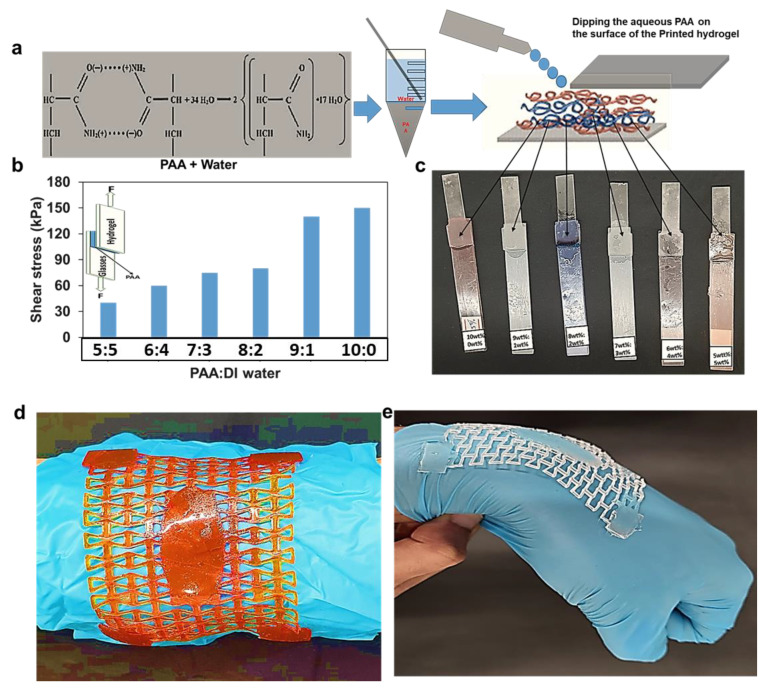
The adhesion strength with different PAA concentrations. (**a**) The PAA and water mixture. (**b**) Shear stress vs. strain for different mixtures of PAA and water. (**c**) Hydrogel sample attached to glass strip using PAA for 24 h. (**d**) The morphological motion of the unit re-entrant phenol red incorporated wound dressings cells when curved in shape. (**e**) The morphological motion of the unit re-entrant cells when the hand joint bends.

Several researchers have used the PAA as adhesion mechanism, either combed with aqueous solutions or pure PAA [39,40,41]. PAA is a biocompatible polymer with abundant carboxyl groups, and they can be dissociated with water [42]. We also developed a PAA- and water-based adhesive for the 3D printed hydrogel wound dressing, using different ratios of PAA: DI water (10:0, 9:1, 8:2, 7:3, 6:4, and 5:5). The adhesion capability of these solutions was experimentally evaluated by performing single-lap shear tests where the PAA-based adhesive was used to bond a 3D printed hydrogel batch to a glass substrate, as shown in Figure 7a. The results show that the shear strength of the lap joint increased with increasing concentration of PAA (Figure 7b). For pure PAA, the shear strength was 148 kPa, while for PAA:water = 1:1, it was only 30 kPa. It should be noted, however, that the adhesive strength between the hydrogel wound dressing and human skin is expected to be less than what is reported here for the glass substrate. We then used the PAA-based adhesive (PAA:water = 7:3) to attach the wound dressing to the wrist joint through four attachment points (Figure 7d–e). A nitrile glove was used for the sake of personal protection. The wound dressing was able to closely follow the motion of the joint without forming wrinkles, thanks to the auxetic behavior of the re-entrant structure.

## 3. Experimental

### 3.1. Materials

2-hydroxyethyl methacrylate C_6_H_10_O_3_ (HEMA), diphenyl (2,4,6-trimethylbenzoyl) phosphine oxide C_22_H_21_O_2_P (TPO), polyethylene glycol dimethacrylate C_10_H_14_O_4_ (PEGDA), isopropyl alcohol (IPA) C_3_H_8_O (Merck, Darmstadt, Germany), acetone C_3_H_6_O (ACS, ISO, Reag. Ph Eur), acrylic acid(AA) C_3_H_4_O_2_, poly(acrylic acid)(PAA) (C_3_H_4_O_2_)n, phosphate-buffered saline C_l2_H_3_K_2_Na_3_O_8_P_2_ (PBS), glass strip, phenol red (0.1% in ca. 20% ethanol) (PR) powder (0.5 g, 0.0014 mol), deionized water (DI water), diluted hydrochloric acid (HCl), pH buffer solutions, ethyl alcohol C_2_H_5_OH (EMSURE^®^ACS, ISO, Reag. Ph Eur).

### 3.2. Hydrogel Resin Preparation and PBS Solution

**Figure 8:**
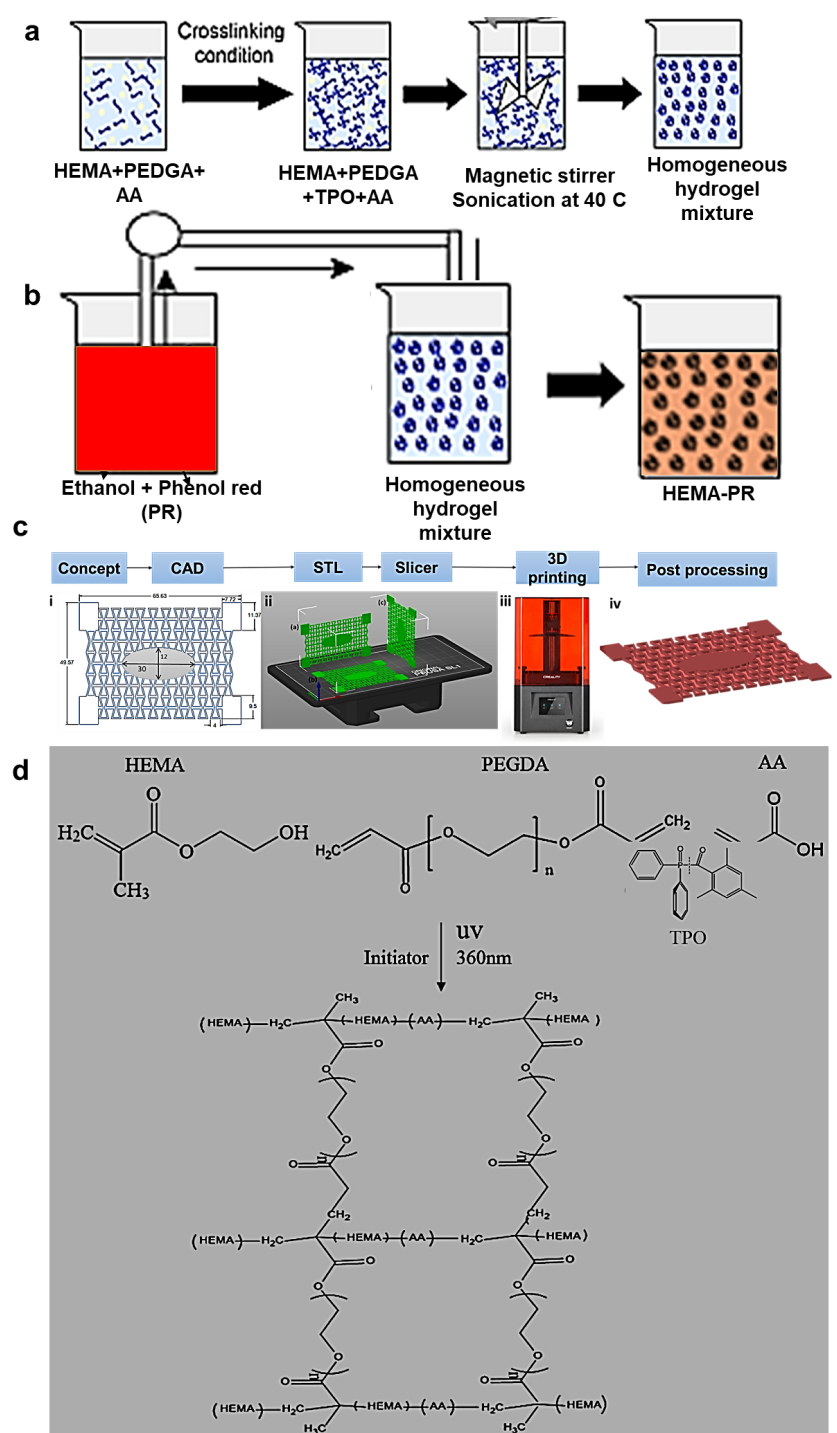
(**a**) Hydrogel resin preparation (**b**) Dissolving of phenol red (PR) in ethanol (**c**) Additive manufacturing process from initial concept to the final printed hydrogel wound dressing (**d**) Chemical synthesis of the *HEMA/PEGDA/TPO/AA*.

A sensitivity analysis on the volume ratios of HEMA monomers, PEGDA crosslinker, and TPO photoinitiator was performed to optimize the mechanical properties of the 3D printed hydrogel. Thus, 20 mL of hydrogel resins were prepared with HEMA (vol.%): PEGDA (vol.%): TPO (wt.%) ratios of 0.5:1:0.1, 1:0.5:0.1, 1:1:0.02, 1:1:0.05, and 1:1:0.1. During the preparation of the hydrogel solution, 5 wt.% of AA was titrated to increase its flexibility (Figure 8a). On the other hand, a solution of 0.1g PR (0.5 gm, 0.00014 mol) and 20 mL of ethanol (C_2_H_5_OH, 46.06844 g/mole) was prepared under homogenous stirring (Figure 8b). After 30 min, the PR dissolved in ethanol completely. The PR-ethanol solution was added to the prepared hydrogel at a ratio of 1:1 by volume to the HEMA based resin. Stirring was performed to disperse the PR in the hydrogel matrix uniformly, and the mixture was heated at 60 °C inside a laboratory oven to evaporate the ethanol from the resin. Ultrasonic baths (frequencies > 20 kHz) were used to remove the bubbles created from the blend of monomers, photoinitiators, and crosslinkers for 25 min.

### 3.3. Preparation of CAD Models

A wound dressing is used to protect a wound, prevent infection, and facilitate healing. A dressing should be large enough to cover the wound, with a margin of at least 2.5 cm on all sides beyond the wound. The 3D geometries of the re-entrant auxetic hydrogel wound bandages were designed using Creo-parametric^®^ (version 3.0) and SolidWorks 2020^®^ (version 28) computer-aided design (CAD) software. The dimensions of the 3D models were considered based on the surface area of the printing bed of Gift Duplicator 7 V1.5 Wanhao SLA DLP 3D Printer^®^ manufactured in United states of America ( USA) with 250 mL resin capacity (see Figure 8c). The wound bandage was designed to cover a small wound on the body joints and had overall dimensions of 66× 50 × 1 mm. Note that the elliptic wound cover at the center of the bandage had dimensions of 30 × 12 mm (see Figure 8c).

### 3.4. 3D Printing Procedures

The *Wanhao D7 WorkShop* slicing software (*version* 1.4) was used to convert the CAD file (in stl format) into a file readable by the 3D printer (also known as G-code). In the slicing software, the initial slice thickness and first exposure time were set to 0.035 mm and 50 s, respectively. After base layers were printed, exposure times were adjusted to 30 s. Since the printing orientation can have a significant effect on the properties of the printed part, three different printing orientations were considered: vertical along the width of the patch, vertical along the length of the patch, and horizontal (Figure 8c(ii)). Once the printing process was completed, the printed parts were taken off the printing platform, and post-processing was applied, as described below. Note that the samples printed with horizontal orientation were selected for the physical and optical characterizations, since they presented the lowest printing time, as well as highest print quality and mechanical strength [1].

### 3.5. Post-Processing

After removing the 3D printed samples from the print bed, they were carefully rinsed with IPA solution to remove the remaining small supports and unreacted monomers. Then, the samples were ultrasonicated in an ultrasonic bath of acetone at 27 °C to remove any remaining uncured polymers. The undesired deep trapped particles were dodged, using sound waves propagated into an IPA-filled vial. The process involves generating alternating high-pressure (compression) and low-pressure (rarefaction) cycles that excite vibrations in the unreacted monomers. In addition, UV post-curing was performed for 2 min using *Legacy UVP Crosslinkers^®^* to ensure complete polymerization of the 3D printed hydrogels. However, excessive UV post-curing could have side effects such as shrinkage, increased stiffness, and reduced water retention capability. We also studied the influence of post-processing parameters (i.e., UV post-curing and ultrasonication) on the mechanical properties of the printed samples.

### 3.6. Characterization

#### 3.6.1. Optical Spectroscopy

Optical spectroscopy was employed to analyze the optical absorption in response to pH changes. It provides information on the strength of phenol red’s pH response in the visible and infrared range. The measurements were performed using a white light source connected via optical fiber to an Ocean Optics spectrometer (Zeiss). Various wavelengths were absorbed (to different degrees) depending on the concentration of the pH solutions.

#### 3.6.2. Shrinkage Behavior

In DLP 3D printing, the photo-polymerization induces linear and volumetric shrinkage of the hydrogels during the printing and curing process. Volumetric shrinkage could occur due to the chemical loss of resin during the photo-polymerization process and thermal contraction during solidification. Typically, the volumetric shrinkage in polymer is less than 3% [43]. The volumetric shrinkage was calculated via:

(1) $$\mathrm{Volume change}(\%)=\frac{V_{3DP\;}-V_{des}}{V_{des}}\times 100$$

where *V_des_* denotes the designed volume of the sample in the CAD software and *V_3DP_* is the volume of the as-printed sample measured using a caliper.

#### 3.6.3. Swelling Capacity

The swelling behavior of the hydrogel was studied according to the report by Lan et al. [43]. The procedure was performed by immersing the 3D printed sample inside a phosphate-buffered saline (PBS) solution. PBS is an isotonic solution in numerous biological research applications [44]. For the purpose of measuring the swelling ratio and degradation rate, one PBS tablet was dissolved in 200 mL of DI water to prepare the PBS solution. The chemical reaction yields 137 mM NaCl, 2.7 mM KCl, 10 mM Na_2_HPO_4_, and 1.8 mM KH_2_PO_4_, at a pH of 7.4 at 25 °C.

Therefore, a 3D printed hydrogel sample (40 × 40 × 1 mm) was weighted (*m*_0_) and immersed in 35 mL PBS solution (pH 7.4) at 25 °C. The swelling capacity of the sample was measured in intervals of 1 h. After removing the samples from the solution, they were wiped with paper tissue and their weight (*m_i_*) was measured. Then, the swelling ratio was calculated as follows:

(2) $$\mathrm{Swelling ratio}(\%)=\;\frac{m_{i}-m_{0}}{m_{0}}\times 100$$

#### 3.6.4. Surface Wettability

Surface wettability measurements were performed using a sessile static drop of 2 µL DI water on the surface of a 3D printed hydrogel. The contact angle (CA) was measured after recording an image of the micro droplet through a camera. A large number of CA measurements were conducted for each droplet, and the mean and the standard deviation (SD) were determined.

#### 3.6.5. Weight Loss (Degradation Rate)

The hydrogel’s degradation was measured as described in H. Baniasadi et al. [31]. The weight loss experiment was carried out by immersing a 3D printed sample (40 × 40 × 1 mm) in 25 mL of PBS solution for 6 days at room temperature (25 °C). The weight of the sample (*m*_0_) was measured prior to immersing it in the PBS solution. The sample was removed after 24 h, and was thoroughly vacuum dried at 40 °C in a laboratory oven for 5 min. The result after 3 days have shown that a new layer starts to degrade and is taken off on day 5. The weight of the sample was measured again (*m_d_*), and the weight loss percentage was calculated as follows:

(3) $$\mathrm{Weight loss}(\%)=\;\frac{m_{0}-m_{d}}{m_{0}}\times 100$$

The same sample was then immersed again in the PBS solution, and the same process was repeated several times to obtain weight loss curves over a period of six days.

#### 3.6.6. Porosity

The temperature-dependent porosity of the 3D printed hydrogel was measured according to the method reported by Baniasadi [31]. Samples measuring 40 × 40 × 1 mm were used for these experiments. First, the weight (*m_i_*) and apparent volume (*V*) of the hydrogel sample were measured. Then, the sample was immersed in 35 mL ethanol until it was fully saturated, and the weight of the saturated hydrogel was measured (*m_sat_*). The porosity (*Ф*) was obtained from Equation (4), in which *ρ* denotes the density of ethanol (0.789 g/mL):

(4) $$Ф(\%)=\;\frac{m_{sat}-m_{i}}{\rho \times V}\times 100$$

To study the effect of temperature on the porosity, the above procedure was conducted in a laboratory oven in the temperature range of 25–50 °C. For this purpose, the hydrogel samples were placed in a laboratory oven for 5 min at various temperature of 25–50 °C and weight measurements were taken. Next they were submerged in the 50 mL of ethanol solution for 1 hr and the final weight was measured. The porosity was calculated according to Equation (4).

#### 3.6.7. Tensile Tests

The tensile stress–strain curves were obtained using a Zwick-Roell Universal Testing Machine (UTM) equipped with a 2.5 kN load cell following standard ASTM D638. All samples were tested to failure under the same environmental conditions (*T* = 25 °C and relative humidity of 60%) using a constant cross-head speed of 2.5 mm min^−1^. The recorded force-displacement data were used to determine the stress–strain response of the tested samples, which allowed deducing basic mechanical properties, such as the yield strength and elastic modulus.

#### 3.6.8. Adhesion Tests

To examine the adhesion capability of the fabricated hydrogel wound dressing, single-lap shear tests were performed as follows. First, a rectangular patch of 3D printed hydrogel (dimensions 10 × 4 × 1 mm) was attached to a glass substrate (dimensions 10 × 4 × 1 mm) through a PAA- and water-based adhesive so as to obtain a single lap joint of width 3 mm. Different PAA-to-water ratios were considered to study potential effects on the adhesive strength. The specimen was then placed between the grips of a Zwick-Roell UTM (2.5 kN load cell) and subject to tensile loading (cross-head speed 1.5 mm min^−1^) until the lap joint failed in shear or teared off the substrate. Note that all shear tests were performed under the same environmental conditions (*T* = 25 °C and relative humidity of 60%). The adhesive strength of the lap joint was then determined via *τ*_max_ = *F*_max_/*A*_joint_, where *A*_joint_ = 12 mm^2^.

## 4. Conclusions

3D printing manufacturing techniques associated with biocompatible hydrogels appeared to be a promising means of developing intricate smart wound dressings that can overcome several traditional bandage fabrication challenges. Additionally, smart wound dressings respond to chemical stimuli, such as pH, ions, and specific chemical compositions. The 3D printed smart re-entrant auxetic hydrogel wound dressing chosen for the study adheres to joint body motion such as knee and elbow movement. Tensile tests revealed a yield strength of 140 kPa and Young’s modulus of 78 MPa. The 3D printed hydrogel wound dressing also shows a swelling capacity of up to 14%, limited weight loss to 3% in six days, and porosity of about 1.2%. Similarly, the study revealed that when the hydrogel polymerization is not completed or exposed to high temperature, it results in porous layered structures. Likewise, the mechanical properties of the printed samples are influenced by post-printing processes, such as ultrasonication used for extracting unreacted monomers, and post-UV curing for further hardening. In addition, we examined the possibility of using phenol red dyes as pH indicators in the hydrogel matrix, which is practically relevant because it could help with monitoring pH changes in the vicinity of the wound. The results showed a range of spectral color changes with various pH solutions. However, the color change on a dry wound is less evident, since the swelling ratio is too low to retain the sufficient amount of pH solution. The main reason behind the leakage of the phenol red dyes could be the insufficient bond formation between the *HEMA/PEGDA/TPO/AA* hydrogel and the phenol red dye (PR) solution. We also developed a PAA based adhesive for the 3D printed hydrogel wound dressing using different PAA-to-water ratios. The highest adhesive strengths in single-lap shear tests were reported when the PAA-to-water ratio was 10:0 and 9:1. In general, water dissociation into the PAA solution was found to weaken the adhesion capability. With the help of the PAA-based adhesive, the auxetic wound dressing was able to closely follow the motion of the wrist joint.

Future research scope could be assessed using adaptable color change dyes that can crosslink with the hydrogel. The swelling capacity of the hydrogel could be modified to obtain adequate pH solution absorption capacity. Specifically, we recommend reinforcing the hydrogels with nanomaterials to enhance their mechanical properties without compromising the swelling capacity. Carbon nanomaterials and graphene oxide have shown great promise in improving the mechanical properties of hydrogels. However, integrating nanomaterials into 3D printable hydrogels remains a challenge which requires further in-depth analysis.

## Figures and Tables

**Table 1:** Mechanical characteristics of 3D printed hydrogels with different proportions of HEMA: PEGDA: TPO.

Polymer Ratios (HEMA:PEGDA:TPO)	Tensile Strength (MPa)	Young’s Modulus (MPa)	Failure Strain (%)
1:1:0.02	2.9	11	2.1
1:1:0.05	4.0	112.5	3.8
1:1:0.1	3.5	78.9	4.5

## Data Availability

The data presented in this study are available on request from the corresponding authors.

## Sample Availability

Samples of the compounds are not available from the authors.
